# High Maternal Neonatal Mortality and Morbidity in Pregnancy with Eisenmenger Syndrome

**DOI:** 10.1155/2021/3248850

**Published:** 2021-09-27

**Authors:** Erry Gumilar Dachlan, Amirah, Nareswari Cininta, Rizky Pranadyan, Alisia Yuana Putri, Yudi Her Oktaviono, Muhammad Ilham Aldika Akbar

**Affiliations:** ^1^Department Obstetrics & Gynecology Faculty of Medicine Universitas Airlangga, Dr. Soetomo General Academic Hospital, Surabaya, Indonesia; ^2^Department Obstetrics & Gynecology Fakfak General Hospital, West Papua, Indonesia; ^3^Department Cardiology Faculty of Medicine Universitas Airlangga, Dr. Soetomo General Academic Hospital, Surabaya, Indonesia; ^4^Department Obstetrics & Gynecology Faculty of Medicine Universitas Airlangga, Universitas Airlangga Hospital, Surabaya, Indonesia

## Abstract

**Objectives:**

This study is aimed at evaluating the maternal and perinatal characteristics and pregnancy outcomes of ES. *Material and Methods*. This is a retrospective cohort study of pregnancy with Eisenmenger syndrome (ES) in Dr. Soetomo Hospital from January 2018 to December 2019. Total sampling size was obtained. We collected all baseline maternal-perinatal characteristic data, cardiac status, and pregnancy outcomes as primary outcomes. The maternal death cases were also evaluated, and we compared characteristics based on defect size (< or >3 cm).

**Results:**

During study periods, we collected 18 cases with ES from a total of 152 pregnancies with heart disease. The underlying heart disease type includes atrial septal defect (ASD), ventricle septal defect (VSD), and patent ductus arteriosus (PDA). All cases suffered pulmonary hypertension (PH), 3 cases moderate, and 15 cases as severe. 94% of cases fall into heart failure (DC FC NYHA III-IV) during treatment. The majority of cases are delivered by cesarean section (88.9%). Pregnancy complications found include preterm birth (78%), low birthweight (94%), intrauterine growth restriction (55%), oligohydramnios (16%), severe preeclampsia (33%), and placenta previa (5.5%). Large defect group has an older maternal ages (30.18 ± 4.60 vs. 24.15 ± 2.75; *p* = 0.002), higher clinical sign (100 vs. 40%, *p* = 0.003), and higher preterm delivery rate (100% vs. 69%, *p* = 0.047) compared to small defect groups. The R to L or bidirectional shunt is significantly higher at the large defect group (13 vs. 5 cases, *p* = 0.006, 95% confidence interval: -1.156 to -0.228). There were seven maternal death cases caused by shock cardiogenic.

**Conclusions:**

Pregnancy with ES is still associated with very high maternal neonatal mortality and morbidity. The larger defect size is correlated with clinical performances and pregnancy outcomes. Effective preconception counseling is the best strategy to reduce the risk of maternal and neonatal death in ES women.

## 1. Introduction

Maternal heart disease is the second common cause of maternal death after preeclampsia. One of the most frequent heart disease types in pregnancy is congenital heart disease in which 5% of the patients have pulmonal hypertension [1]. Eisenmenger syndrome (ES) represents the severe end of the spectrum for disease in pulmonary arterial hypertension associated with congenital heart defect (PAH-CHD). Eisenmenger syndrome due to PAH-CHD is classified into group one. It is defined by systemic-to-pulmonary shunting of blood through any large congenital cardiac defects at any location permitting increased pulmonary blood flow that progress into severe elevation of pulmonary vascular resistance (PVR) resulting in reversal (pulmonary-to-systemic) or bidirectional shunting [2]. The defect is considered inoperable. Hemodynamically, ES is defined as the elevation of PVR to 12 Wood unit or to a pulmonary-to-systemic resistance ratio equal to or greater than 1.0. The size of shunt and exact diameter of the defect do matter. The threshold is 2-3 cm at atrial level, 1-1.5 cm at ventricular level, and 0.5-0.7 cm at arterial level. Fifty percent of patients with a large defect in the ventricular level developed ES, and 13% percent of patients with large defect at the atrial level develop ES [3].

The prevalence of ES is not well-known. Recent data showed that 4.2% of adult CHD patients develop PAH-CHD, and one percent had ES. The incidence of ES is even lower in pregnancy, approximately around 3% in pregnant women with congenital heart disease [4]. Patients with ES who become pregnant have a very high risk of adverse pregnancy outcomes. Maternal mortality is reported 30-50%, and most of it is caused by rapidly progressive cardiopulmonary decompensation, thrombotic complications, and sudden death due to malignant arrhythmia [5]. Because of the high risk of maternal mortality, pregnancy is contraindicated in women with ES [6]. Adverse pregnancy outcomes found in ES are abortion, intrauterine growth restriction/IUGR (30%), and preterm birth (50-60%) which are all related to maternal chronic hypoxia [7]. Eisenmenger syndrome is often diagnosed and managed late, especially in developing countries. This is related to the low awareness and knowledge in the community and social and economic factors that delay access to public health services [8]. This study is aimed at evaluating the maternal, perinatal, and pregnancy outcomes of ES.

## 2. Materials and Methods

### 2.1. Study Population and Outcomes

This is a retrospective cohort study of pregnancy with ES cases at Dr. Soetomo General Academic Hospital, Surabaya, East Java, Indonesia, from January 2018 to December 2019. Dr. Soetomo General Academic Hospital is the top referral tertiary center hospital in East Java, Indonesia. The study population was pregnant women with heart disease who were managed in our hospital during study periods. The inclusion criteria were all pregnant or postpartum women with ES. There are no exclusion criteria in this study. We used a total sampling size. The ethical clearance of this study was approved by the Ethical Committee Board of Dr. Soetomo Hospital Written informed consent has been acquired from all the participants. The primary outcomes of this study are maternal, perinatal, and pregnancy outcomes in Eisenmenger syndrome. Pregnancy outcomes evaluated consist of maternal mortality, obstetrics complications (abnormal cardiotocography test, oligohydramnios, severe preeclampsia, and IUGR), heart failure, preterm delivery rate, mode of delivery, baby birthweight, and Apgar score. We also assessed the maternal characteristics and the maternal cardiac status. Maternal characteristics include maternal ages, gestational age at diagnosis and delivery, gravidity, antenatal care history, heart disease type, and heart failure. Cardiac status includes heart disease type, murmur sign, clubbing finger, arterial oxygen saturation (SaO_2_), defect size, and pulmonary arterial systolic pressure (mmHg). The defect size and pulmonary arterial systolic pressure were determined using echocardiography examination, while the other cardiac parameters were found from the physical examination. We also evaluated the correlation between maternal and perinatal characteristics and pregnancy outcomes with the cardiac defect size. We divided the samples into two groups based on the defect size: small (<3 cm) and large (>3 cm) defect groups. The clinical characteristics of all maternal death cases were also described.

We defined abnormal cardiotocography as a finding of category two or three based on National Institute of Child Health and Human Development criteria [9]. Oligohydramnios was diagnosed as an amniotic fluid index < 5 cm, from fetal ultrasound [10]. We defined preeclampsia as gestational hypertension accompanied by >1 of the following new-onset conditions after 20-week gestation: proteinuria, maternal organ dysfunction, or uteroplacental dysfunction [11]. IUGR was defined based on ultrasound finding of estimated fetal weight < 10 percentile [12]. Heart failure was categorized based on the New York Heart Association (NYHA) functional class [13]. Preterm was defined as delivery before 37-week gestation [14–16]. Apgar score was used to evaluate general health and sign of hemodynamic compromise of the newborn. Score < 7 was defined as low Apgar scores [17].

### 2.2. Echocardiography Procedure

Transthoracic echocardiography (TTE) was performed by adult cardiology fellows. All the procedures were supervised, and all the results are discussed by the senior cardiologist from echocardiology and adult congenital heart disease division to minimize interoperator variation bias. The heart scanning machine was Echo Vivid E9 GE and EchoPAC Dimension system (General Electric Healthcare, US) with the procedure as follows:

1. All TTE parameters were examined based on American Society of Cardiography (ASE) recommendation
2. Two-dimensional modes were scanned from all views, including parasternal long axis (PLAX), parasternal short axis (PSAX), apical 4-chamber, apical 2-chamber, subcostal, and suprasternal
3. Pulse-wave (PW) Doppler and colour-wave (CW) Doppler mode were scanned from all views to detect any cardiac lesion and shunts
4. Left ventricle (LV) systolic function was estimated using *Teichholz* methods from M-mode in PLAX and also using modified *Simpson's* methods in an apical 4-chamber and 2-chamber view
5. Left ventricle (LV) diastolic function was measured using PW Doppler and tissue Doppler imaging (TDI) in a 4-chamber view
6. Probability of pulmonal hypertension related to congenital heart defect was estimated by the addition of tricuspid regurgitation maximal pressure gradient (TR max. PG) and estimated right atrial pressure (est. RAP). Estimated pulmonal artery pressure (Est. PASP) is also assessed from peak velocity of tricuspid regurgitation (TR V max) and other PH signs from the ventricle, pulmonary artery, inferior vena cava, and right atrium

### 2.3. Statistical Analysis

The data of maternal and perinatal characteristics were evaluated using descriptive analysis. The categorical variables were compared between groups using the chi-square test or Fisher exact test based on its distribution. The numerical variables were compared using an independent *t*-test. All statistical tests were performed using IBM Statistics SPSS 25.

## 3. Results

### 3.1. Maternal and Perinatal Characteristic

During study periods, we collected a total of 152 pregnant and postpartum women with heart disease. Among them, there are 18 cases (11.8%) with ES that consisted of fifteen pregnancies (83%) and three postpartum cases (17%). Most cases (90.5%) were identified as ES on admission to the hospital. The maternal age average was 27.17 years old, and most are in the interval of 20-29 years old (83%). The majority of cases were primigravida (61%). Fifteen (83%) cases were referred from lower-level hospital/public health services. Only 17% cases had a regular antenatal care in our hospital and were managed by a multidisciplinary team until delivery (Table 1). Most patients were first diagnosed as having ES in the gestational ages of 28-36 weeks (61%). The heart diseases found in the study were atrial septal defect (ASD), ventricle septal defect (VSD), and patent ductus arteriosus (PDA) (Table 1).

Pregnancy complications found include preterm birth (78%), low birthweight (94%), intrauterine growth restriction (55%), oligohydramnios (16%), severe preeclampsia (33%), and placenta previa (5.5%). The majority of cases are delivered by cesarean section, and newborns have a low birthweight related to preterm birth. Most cases are straightly sterilized during or after delivery (72%) (Table 1).

All cases suffered pulmonary hypertension (PH), which was divided into the moderate (3 cases) and severe type (15 cases). Moderate PH is defined by the pulmonary artery systolic pressure (PASP) 60-80, and the PASP in severe PH is >80. Echocardiography results show a common defect size of 1-2 cm in PDA (1 case) and VSD cases (4 cases). Defect 2-3 cm (7 case) and large defect > 3 cm (5 case) are only found in ASD cases. The shunting pattern in the heart was commonly right to left (10 cases), followed by bidirectional (5 cases) and left to right (3 cases) flow (Table 2). Unfortunately, most cases fall into heart failure during gestation (94%), including NYHA classification DC-DC III and DC-FC IV. Laboratory parameters, including hemoglobin (Hb), leukocyte, platelet count, hematocrit (Hct), and oxygen saturation, can be seen in Table 2. The mean value of each parameter was as follows: Hb: 13.9 g/dL, Hct: 41.68%, leukocyte: 10.971 cell/*μ*L, platelet count: 200.667 cell/*μ*L, and oxygen saturation: 88%. Most patients have a normal Hb levels, and only two women have anemia (Hb < 11 g/dL). There is only one woman who has an abnormal leukocyte count (>17.000) and four women with thrombocytopenia (<150.000). Only five cases have a normal oxygen saturation during admission (≥95%), while the others are already in a hypoxia state. Seven cases even show a sign of respiratory failure, with an oxygen saturation < 85% (Table 2).

### 3.2. Relationship between Maternal-Perinatal Characteristic and Cardiac Defect Size

We divided all cases into two groups based on the defect size in the heart, including small defect (<3 cm) and large defect (≥3 cm) groups. All maternal and perinatal characteristics are compared among these two groups. Maternal ages in the large defect group are significantly older than the small defect group (30.18 ± 4.60 vs. 24.15 ± 2.75). All cases in the large defect group show a clinical sign of clubbing finger and cyanosis; however, only forty-six percent manifest in the small defect group. Regarding the heart disease type, the large defect group consisted of ASD (100%), while the small defect has an ASD, VSD, and PDA. All cases in the large defect group end in preterm delivery, compared to sixty-nine percent in the small defect group. All other parameters show no significant difference statistically (Table 3).

### 3.3. Relationship between Defect Size, Shunt Type, and Pulmonary Hypertension

This study evaluated the relationship between pulmonary hypertension, R to L or bidirectional shunt, and defect size. The small defect group (≤3 cm) showed a significantly higher proportion of PH compared to large defect (13 vs. 5 cases, *p* = 0.037, 95% confidence interval: 1.040 to -0.0.37), but no correlation was found between both parameters (*p* = 0.063). The R to L or bidirectional shunt is significantly higher at the large defect group (13 vs. 5 cases, *p* = 0.006, 95% confidence interval: -1.156 to -0.228). Contingency coefficient correlation test resulted in *r* = 0.527, indicating a strong correlation between bidirectional or R to L shunt with defect size.

### 3.4. Maternal Death Case

We found seven maternal death cases in this study (38.8%), and all happened in ASD cases. The cause of death in these cases is a cardiogenic shock after delivery. All cases were lately referred to our hospital in the third trimester in an already deteriorated condition. All cases are complicated by severe PH, three cases by severe preeclampsia, and 1 case by cardioembolic stroke. All maternal deaths have happened in less than two weeks after delivery; the fastest was 16 hours after delivery. The clinical characteristics of maternal death can be seen in Table 4.

## 4. Discussion

ES is an acquired elevation of pulmonary vascular resistance and pulmonary artery pressure due to a left-to-right intracardiac shunt. These pathological changes lead to a reversal right-to-left or bidirectional shunt, with subsequent cyanosis and polycythemia. As shown in Table 3, over two years, we found most cases are severe PH (89%), which are dominated by right to left shunt (55.6%). However, most PHT cases arise from the small defect group (72.2%). In this study, most ES patients come from rural residents in East Java Province, with low socioeconomic-educational level and poor physical status. They got a chronic tolerance to cardiac heart disease by no symptoms over a long period, which eventually manifests in pregnancy. Due to a lack of general and medical knowledge, these people lack access to health services, especially cardiologists. The majority refused the advice of cardiac surgery on the patients who can receive cardiac health services. The reason is lack of awareness of the increased risk of morbidity and mortality of pregnancy with cardiac disease and its associated ES complications. There is also a lack of preconceptional counseling nor antenatal care with the obstetrician and cardiologist before pregnancy [18].

The clinical manifestation of ES (clubbing finger or cyanosis) also significantly correlated with the defect's size. The larger the defect size, the higher the possibility of clinical signs appearing. The defect size also correlates with the disease's risk of progression, which is shown by the reversal flow pattern in the heart shunt (R to L or bidirectional shunt). The reversal or bidirectional shunt is found prominently in our large defect group compared to the small one. In ES, especially large septal defects, lesions are characterized by high pulmonary pressure and a high pulmonary flow state. ES refers to any untreated congenital cardiac defect with intracardiac communication that leads to pulmonary hypertension, reversal of flow, and cyanosis. The previous left-to-right shunt is converted into a right-to-left shunt secondary to elevated pulmonary artery pressures and associated pulmonary vascular disease [19, 20].

El Kayam et al. explained, eventually, that due to increased resistance and decreased compliance of the pulmonary vessels, elevated pulmonary pressures cause the right heart myocardium hypertrophy (RVH). ES begins when RVH causes right heart pressures to exceed the left heart pressure, leading to a reversal of blood flow through the shunt. Consequently, deoxygenated blood returning from the body bypasses the lungs through the reversed shunt and directly to the systemic circulation, leading to cyanosis and resultant organ damage [20].

In pregnant women, the congenital heart diseases that cause pulmonary vascular disease and evolve into ES are mainly VSD, followed by ASD and PDA. Pregnant women with ES may present with clubbing fingers, cyanosis, dyspnea, fatigue, dizziness, or even right heart failure. This study shows that cyanosis and clubbing fingers and cardiac septal lesion were more prominent in larger defect sizes (> 3 cm). Blood gas analysis, complete blood count, and oxygen saturation are important factors in pregnant women with ES. Previous studies showed that oxygen saturation < 65%, Hct > 60%, and Hb > 18 g/dL are predictors of adverse maternal outcomes in pregnancy [21, 22]. The majority of this study's cases have oxygen saturation < 85%, with the lowest saturation which is 66%. The mean hematocrit value in this study is relatively high (42%) compared to a similar study from India (35.3%) [23]. Most cases show polycythemia (Hb > 16 g/dL), indicating chronic hypoxia (Table 2). Pregnant women with ES should be hospitalized after the 20th week of pregnancy—or earlier if clinical deterioration occurs [20, 24–26]. A person with ES is paradoxically subject to the possibility of both uncontrolled bleeding due to damaged capillaries and high pressure and spontaneous clots due to hyperviscosity and blood stasis [27].

ES in pregnancy can cause severe complications, although successful delivery has been reported. Maternal mortality ranges from 30% to 60% and may be attributed to heart failure, venous thromboembolic event, hypovolemia, or cardiogenic shock. Six cases have a defect size > 2 cm, and even three of them have >3 cm defect. Most deaths occur either during or within the two weeks after delivery. Study shows that 10% of women with ES are deceased within 14 days after delivery. Even in ES related to congenital heart disease, this number increases by 28%, and the average survival time is only six days [28]. All maternal death in this study is caused by cardiogenic shock and suffered a severe PH. This study's most maternal death has happened less than two weeks after delivery, related to redistribution of fluid in postpartum periods. This volume overload will exceed the heart capacity with a defect and lead to complications such as heart failure, atrial fibrillation, pulmonary edema, or shock cardiogenic. Katsurahgi et al. study mentioned that from 73 cases of ES, the majority of death was on postpartum periods compared to antepartum (23 vs. 3 cases) [29]. Three maternal death cases coincide with severe PE, which complicated the hemodynamic changes in patients with ES. Maternal mortality in heart disease is still high three until four weeks after delivery [30]. The first two-week postpartum is the highest risk of maternal death in pregnancy with heart disease. This finding may suggest the need to prolong women with heart disease in the intensive care unit after delivery. All maternal death cases come in an already severe condition related to the late referral. All cases are already complicated by severe PH, heart failure (DC FC NYHA III-IV), and low oxygen saturation. Although a multidisciplinary team has managed patients in the ICU and the pregnancy was immediately terminated, the disease's progression cannot be stopped. All patients died <10 days after delivery.

Most patients delivered by cesarean section (88%). Only two women deliver vaginally; both are in nonviable and periviable gestational age. One case is in pregnancy with severe PH in the first trimester (12 weeks), which is terminated due to an increased risk of maternal mortality if the pregnancy is continued. The woman is 24 years old and not aware of her congenital heart disease before pregnancy. The other case is the first pregnancy on 27-week gestation and complicated by perimembranous VSD, moderate PH, and ES. Delivery is indicated due to the worsening condition of the mother and born 800-gram babies with a low Apgar score. Unfortunately, this mother passed away 48 hours after delivery. Although there is no evidence of a superior mode of delivery in heart disease, cesarean delivery is preferable in case of severe heart disease with a poor maternal condition such as ES. Vaginal delivery benefits from lower blood loss, risk of thromboembolism, and risk of infection compared to cesarean section. However, vaginal delivery should be performed with a device (forceps extraction or vacuum) to accelerate the second stage in a term gestation [31]. Our hospital's preferred delivery model is decided by the multidisciplinary team, including obstetricians, intensivists, anesthesiologists, cardiologists, and perinatologists. In the most condition of ES, the cesarean is favorable due to the faster procedure, more tight monitoring, and more controllable maternal condition. In cesarean delivery, the mother also does not have to face the uterine contraction, which massively increases cardiac output and heart failure risk [21].

The outcomes of pregnant women with ES in this study are poor. Ninety-four percent (17 cases) of babies were born in preterm gestation with birthweight < 2500 grams. Ten babies were confirmed to have an IUGR condition after being born from the Ballard-Lutchenzko score [32]. ES in pregnancy is a significant risk factor of IUGR, and the majority deliver in preterm gestation [2, 4, 26, 29]. However, the prevalence of IUGR in pregnancy with corrected congenital heart disease (without severe residual defects) is only 8 out of 50 pregnancies (16%) [33]. Another study shows that neonatal outcomes in the corrected heart are much better compared to the uncorrected, including prematurity (0 vs. 40%), IUGR (20% vs.40%), neonatal death (0 vs. 10%), and baby birthweight (2.17 kg vs. 1.62 kg) [34]. Unfortunately, in this study, all cardiac lesions are uncorrected for the reason that was already explained. The neonatal complication found includes respiratory distress syndrome (9 cases), early-onset sepsis (1 case), and necrotizing enterocolitis (1 case).

## 5. Conclusions

Pregnancy with ES, incredibly complicated by PH, is still associated with very high maternal and fetal morbidity and mortality. Effective preconception counseling is essential to correct heart disease before pregnancy. Termination of pregnancy in the first trimester is advisable in severe heart diseases such as ES and PH. Maternal risks would increase significantly if the pregnancy continued until the third trimester. Suppose the ES patient with PH wishes to carry on the pregnancy. In that case, they should be monitored closely and managed in a tertiary center with collaborative efforts among obstetricians, cardiologists, anesthesiologists, pediatricians, and intensivists. There is no standardized approach to the management of ES in pregnancy; successful perinatal outcomes seem heavily dependent on each patient's individualization of treatment.

## Figures and Tables

**Table 1:** Maternal characteristics and pregnancy outcomes of Eisenmenger syndrome.

Maternal age (yrs)	Patient numbers *n* (%)
20-29 ≥30	15 (83) 3 (17)
Maternal age mean (year)	27.17
Gestational age on diagnosis (weeks)	
<20	1 (5.5)
20-27	2 (11)
28-36	11 (61)
37-40	1 (5.5)
Postpartum	3 (17)
Gestational age at delivery (weeks)	
Preterm (<37)	14 (78)
Term (≥37)	1 (5)
Postpartum	3 (17)
Gravidity	
Primigravida	11 (61)
Multigravida	7 (39)
Antenatal care	
NBC	15 (83)
BC	3 (17)
Heart disease type	
ASD	13 (73)
VSD	4 (22)
PDA	1 (5)
Heart failure	
No heart failure	1 (6)
DC FC NYHA III	9 (50)
DC FC NYHA IV	8 (44)
Obstetric complications	
Abnormal CTG	2 (11)
Severe preeclampsia	9 (50)
Placenta Previa	2 (11)
Oligohydramnios	2 (11)
IUGR	9 (50)
Mode of delivery	
Cesarean section	16 (88.9)
Vaginal delivery	2 (11.1)
Contraception methods	
Sterilization	13 (72)
IUD	1 (6)
No contraception	3 (20)
Baby birthweight	
<2500 gram	17 (94)
≥2500 gram	1 (6)
Apgar score	
1-3	3 (20)
4-6	3 (20)
7–9	9 (60)

NBC: nonbooked case; BC: booked case; ASD: atrial septal defect; VSD: ventricle septal defect; PDA: patent ductus arteriosus; DC FC NYHA: Decompensatio Cordis Functional Class New York Heart Association; CTG: cardiotocography; IUGR: intrauterine growth restriction; IUD: intrauterine device.

**Table 2:** Cardiac status and laboratory parameters of Eisenmenger syndrome.

Case	Heart disease	Murmur	Clubbing finger	SaO_2_ (%)	Defect size (cm)	PASP (mmHg)	Hb (g/dL)	WBC (/*μ*L)	PLT (/*μ*L)
1	ASD	Y	N	97	3	80.37	8.1	16,460	181,000
2	ASD	Y	N	83	3	91.24	15.5	11,400	284,000
3	VSD	N	Y	84	1,1	45.35	18.1	8,180	125,000
4	VSD	Y	Y	88	1	76	18.6	9,930	100,000
5	ASD	Y	N	95	2.3	69.25	11.1	7,220	200,000
6	ASD	Y	N	69	3.7	133.50	19.3	9,520	142,000
7	ASD	Y	Y	84	3.9	77.28	15.1	9,640	206,000
8^∗^	ASD	Y	Y	89	2.9	181.66	14.4	8,670	151,000
9^∗^	ASD	Y	N	95	2.1	120.50	13.7	11,360	269,000
10^∗^	ASD	Y	N	97	3.0	48.58	14.2	12,000	200,000
11^∗^	ASD	Y	N	91	3.2	130.43	13.7	11,640	256,000
12^∗^	VSD	Y	N	83	1.4	111	13.5	9,090	274,000
13^∗^	ASD	Y	Y	78	3.1	144.89	13	7,840	175,000
14	ASD	N	N	94	2.4	117.42	12.7	10,350	202,000
15	VSD	Y	N	90	0.7	45.34	11.8	13,650	307,000
16	PDA	Y	N	95	1.4	115.67	7.9	23,530	142,000
17	ASD	Y	N	81	3.4	102.79	14.3	6,480	199,000
18^∗^	ASD	Y	Y	89	1.7	86.43	15.8	10,520	199,000

∗ indicates maternal death cases. ASD: atrial septal defect; VSD: ventricular septal defect; PDA: patent ductus arteriosus; Y: yes; N: no; SaO_2_: arterial oxygen saturation; PASP: pulmonary artery systolic pressure; Hb: hemoglobin; WBC: white blood cell; PLT: platelet count.

**Table 3:** Relationship between maternal perinatal characteristic and cardiac defect size.

Characteristics	Lesion diameter of <3 cm *n* (%)	Lesion diameter of ≥3 cm *n* (%)	Total (*n* = 18)	*p*
Maternal age (yrs)				
20–29	12 (92)	3 (60)	15 (83)	0.266
≥30	1 (8)	2 (40)	3 (7)	
Maternal age (yrs)	24.15 ± 2.764	30.18 ± 4.604		0.002^∗^
GA at labor				
Preterm	9 (69)	5 (100)	14 (72)	0.047^∗^
Term	1 (8)	0 (0)	1 (6)	
Postpartum	3 (23)	0 (0)	3 (12)	
Gravida				
Primigravida	9 (69)	2 (40)	11 (61)	0.281
Multigravida	4 (31)	3 (60)	7 (39)	
ANC				
NBC	10 (77)	5 (100)	15 (83)	0.082
BC	3 (23)	0 (0)	3 (17)	
Clubbing finger & cyanosis				
Negative	7 (54)	0 (0)	7 (38)	0.003^∗^
Positive	6 (46)	5 (100)	11 (62)	
Hemoglobin level				
<16 g/dL	11 (85)	2 (40)	13 (72)	0.063
≥16 g/dL	2 (15)	3 (60)	5 (28)	
Hematocrit				
<40%	6 (46)	1 (20)	7 (38)	0.318
≥40%	7 (54)	4 (80)	11 (62)	
SaO_2_				
<90%	11 (85)	4 (80)	15 (83)	0.827
≥90%	2 (15)	1 (20)	3 (17)	
Heart disease type				
ASD	8 (62)	5 (100)	13 (72)	0.027^∗^
VSD	4 (30)	0 (0)	4 (22)	
PDA	1 (8)	0 (0)	1 (6)	
Delivery methods				
Cesarean section	11 (85)	5 (100)	16 (89)	0.165
Vaginal delivery	2 (5)	0 (0)	2 (11)	
Baby birthweight				
<2,500 g	12 (92)	5 (100)	17 (94)	0.552
≥2,500 g	1 (8)	0 (0)	1 (6)	
Apgar score				
1-3	2 (20)	1 (20)	3 (20)	0.968
4-6	1 (10)	2 (40)	3 (20)	
7–9	7 (70)	2 (40)	9 (60)	

∗ indicates a significant value (*p* < 0.05). GA: gestational age; ANC: antenatal care; NBC: nonbooked case; BC: booked case.

**Table 4:** Clinical characteristics of maternal death case.

No.	Age (year)	Gravida	Possible cause of death time	Cardiac lesion	Cardiac complication	Obstetric complication	Delivery method
1	24	Primigravida	Cardiogenic shock/16 hrs after CS	ASD-R to L	Severe PHT DCFC IV	IUGR fetus	CS + tubectomy bilateral
2	35	Multigravida	Cardiogenic shock/8 days after CS	ASD-R to L	Severe PHT Lung Oedema	Severe preeclampsia Oligohydramnios	CS + tubectomy bilateral
3	20	Primigravida	Cardiogenic shock/7 days after CS	ASD-R to L	Severe PHT	Severe preeclampsia Complete placenta previa	CS + tubectomy bilateral
4	35	Multigravida	Cardiogenic shock/5 days after CS	ASD-R to L	Severe PHT Cardioembolic stroke	—	CS + tubectomy bilateral
5	24	Primigravida	Cardiogenic shock/17 hours after CS	ASD-R to L	Severe PHT DCFC IV	Severe preeclampsia	CS + tubectomy bilateral
6	24	Multigravida	Cardiogenic shock/4 days after delivery	ASD-R to L	Severe PHT	Underweight (body mass index = 16) IUGR fetus	Vaginal delivery
7	25	Primigravida	Cardiogenic shock/10 days after CS	ASD-R to L	Severe PHT	Underweight (body mass index = 17) IUGR fetus	CS + tubectomy bilateral

## Data Availability

The data is available on request via the institutional board of Dr. Soetomo General Academic Hospital. The authors do not own the data. The data right is held by Dr. Soetomo General Academic Hospital.
